# Compositional and functional alterations in the oral and gut microbiota in patients with psychosis or schizophrenia: A systematic review

**DOI:** 10.12688/hrbopenres.13416.1

**Published:** 2021-09-29

**Authors:** Nuala Murray, Sukainah Al Khalaf, David Kaulmann, Edgar Lonergan, John F Cryan, Gerard Clarke, Ali Khashan, Karen O’Connor

**Affiliations:** 1 Department of Psychiatry and Neurobehavioural Science, University College Cork, Cork, T12XF62, Ireland; 2 School of Public Health, University College Cork, Cork, T12XF62, Ireland; 3 INFANT Research Centre, University College Cork, Cork, T12XF62, Ireland; 4 RISE, Early Intervention in Psychosis Service, South Lee Mental Health Services, Cork, Ireland; 5 Department of Anatomy and Neuroscience, University College Cork, Cork, T12XF62, Ireland; 6 APC Microbiome Ireland, University College Cork, Western Rd, Cork, Ireland T12XF62, University College Cork, Cork, T12XF62, Ireland

**Keywords:** Schizophrenia, Psychosis, Microbiota, Microbial Metabolites, Inflammation, Diversity

## Abstract

**Background: **Gut and oral microbiota are intrinsically linked to human health. Recent studies suggest a direct link with mental health through bidirectional gut–brain pathways. Emerging evidence suggests that the composition and/or function of intestinal microbiome differs in those with psychosis and schizophrenia as compared with controls. There is relatively little research on the predicted or actual functional alterations associated with the composition of oral and gut microbiota in patients with psychosis. We will perform a systematic review and meta-analysis to identify, evaluate and if possible, combine the published literature on compositional alterations in the oral and gut microbiota in patients with psychosis or schizophrenia compared with healthy controls. We also aim to explore the potential functional impact of any compositional changes.

**Methods: **Original studies involving humans and animals using a case-control, cohort or cross-sectional design will be included. The electronic databases PsycINFO, EMBASE, Web of Science, PubMed/MEDLINE and Cochrane will be systematically searched. Quantitative analyses will be performed using random-effects meta-analyses to calculate mean difference with 95% confidence intervals.

**Discussion: **Changes in microbiota composition in psychosis and schizophrenia have been correlated with alternations in brain structure and function, altered immunity, altered metabolic pathways and symptom severity. Changes have also been identified as potential biomarkers for psychosis that might aid in diagnosis. Understanding how predicted or actual functional alterations in microbial genes or metabolic pathways influence symptomatic expression and downstream clinical outcomes may contribute to the development of microbiome targeted interventions for psychosis.

**Registration: **The study is prospectively registered in PROSPERO, the International Prospective Register of Systematic Reviews (CRD42021260208).

## Introduction

The gut microbiota is a complex ecological community of microbes which are diverse and personalized ^1^ and includes bacteria, fungi and viruses ^2^. The collective microbial genome of these microorganisms is termed the microbiome and is often considered a second modifiable genome in the human body ^3^. This gastrointestinal ecosystem is intrinsically linked to human health and recent studies suggest a direct link with mental health ^4^. Gut microorganisms communicate with the brain through the gut-brain axis in a two-way communication system ^5, 6^. This axis describes key pillar physiological systems including the endocrine, nervous, immune, and metabolic systems facilitating behavioural responses ^7, 8^. Several processes have been investigated as mechanisms underpinning communication along this axis, including tryptophan metabolism ^9, 10^, the hypothalamic-pituitary-adrenal axis ^11^, the production of microbial metabolites such as short chain fatty acids ^12^ and the vagus nerve ^6^. A healthy microbiome supports effective signalling along these bidirectional gut–brain pathways ^13^. These pathways may impact on a broad range of neurological and psychiatric conditions. Compositional and functional microbiome alterations have been associated with central nervous system (CNS) disorders including depression ^14, 15^, Parkinson’s disease ^16^, autistic spectrum disorder ^17, 18^, and attention deficit hyperactivity disorder ^19, 20^. Emerging evidence suggests that the composition and/or function of intestinal microbiome differs in those with schizophrenia as compared with controls ^21– 23^.

Psychosis is a condition that affects the way the brain processes information. When experiencing psychosis people can become disconnected from reality and experience hallucinations and delusions. They can experience social withdrawal, anhedonia, and struggle to motivate themselves. Impaired cognition is also a typical symptom of acute psychosis. Psychosis is associated with an array of mental illnesses including schizophrenia, schizoaffective disorder, delusional disorder, bipolar disorder and depression. Psychotic symptoms can also be drug induced.

The aetiology of psychosis is thought to be multifactorial and the result of interactions between genetic and environmental factors ^24^. Several theories have attempted to explain its pathogenesis and alterations in neurotransmitter pathways involving dopamine, glutamate, and γ-aminobutyric acid (GABA) have been implicated ^25– 27^. Converging evidence suggests that psychosis is associated with chronic systemic and gastrointestinal inflammation, oxidative stress, and metabolic dysfunction ^28^. Metabolic and gut disturbances are highly prevalent in psychosis and schizophrenia, with comorbidities including celiac disease, colitis, and irritable bowel syndrome ^29^. In fact, gastrointestinal diseases are the third leading cause of natural deaths in schizophrenia ^30^. Physiological dysfunctions implicated in psychosis such as inflammation and oxidative stress may be associated with changes in the microbiome ^21^.

Whilst empirical investigations have focused on elucidating compositional and functional aspects of the intestinal microbiota in maintaining ongoing physiological processes within the gastrointestinal tract ^31, 32^, there is relatively little research on the predicted or actual functional alterations associated with the composition of oral and gut microbiota in patients with psychosis. Multiple studies have documented an interaction between the gut microbiome and immunity, cognitive functioning, and behaviour in animal models. For example, findings from mechanistic studies in rodent models demonstrate that compositional alterations during early neurodevelopment or in adulthood can lead to direct CNS effects that manifest during adulthood ^33, 34^. Translational support from clinical populations that reflect endophenotypes characteristic of schizophrenia is more difficult to obtain. We will perform a systematic review and meta-analysis to identify, evaluate and if possible, combine data on the compositional alterations in the oral and gut microbiota in patients with psychosis or schizophrenia. We also aim to explore mechanistic insights and/or the evidence for a potential causal role and in both human and animal models. 

## Objectives

1. To systematically review previous studies that have investigated the association between psychosis or schizophrenia and compositional and functional alterations in the oral and gut microbiota.
2. To undertake a meta-analysis of compositional alterations in the oral and gut microbiota in patients with psychosis or schizophrenia.
3. To systematically review the data on the clinical and functional consequences of these alterations in humans and animals, from a causal and mechanistic perspective.

## Methods

The protocol for this systematise review was registered on PROSPERO the international prospective register of systematic reviews (CRD42021260208). This systematic review and meta-analysis will be conducted in accordance with the guidelines for systematic review and meta-Analysis (PRISMA) and protocols (PRISMA-P) (online supplemental appendix 1) ^35^.

## Eligibility criteria

The systematic review will include original studies using a case-control, cohort or cross-sectional design. Case reports, expert opinions, and reviews will not be eligible for inclusion. Clinical studies will include human adults. Preclinical studies will focus on animal studies involving the faecal microbiota transplant of psychosis-associated microbiota consortia.

## Information sources and search strategy

The electronic databases PsycINFO, EMBASE, Web of Science, PubMed/MEDLINE and Cochrane will be systematically searched. Peer-reviewed research articles from 1990–2021 will be considered, so that current literature is evaluated. The search terms will include both free text and MeSH terms. Our search strategy for PubMed/MEDLINE is as follows:

1. (‘microbiota’ OR ‘microflora’/ OR ‘microbiome’ OR ‘bacterial microbiome’/ OR ‘intestine mucosa’/ OR ‘gut brain’ OR ‘oral microbiota’ OR ‘oral microbiome’/ OR ‘oral microflora’/ OR ‘RNA 16S’/ OR ‘gut microbiota’ OR ‘gut microbiome’ OR ‘gut microflora’/ OR ‘gut bacteria’/ OR ‘gut dysbiosis’ OR ‘phageome’ OR ‘faecal’ OR ‘faecal microbiota transplant*’ OR ‘intestinal microbiota transfer’/ OR ‘faeces infusion’ OR ‘donor faeces infusion’/)

2. (‘alpha diversity’ OR ‘beta diversity’ OR ‘human’/ OR ‘animal’/ OR ‘intestine flora’/ OR ‘microbial diversity’/ OR ‘taxonomy’ OR ‘metagenom*’ OR ‘microbiota’ OR ‘gut microbiome composition’ OR ‘metabolomic’/ OR ‘compositional alteration*’ OR ‘functional alteration*’ OR ‘brain function’ OR ‘symptom severity’ OR disease severity’/ OR ‘behaviour’ OR ‘functional pathway’)

3. Searches 1 and 2 were then be combined (1 ‘OR’ 2)

4. (‘schizophrenia’ OR ‘psychosis’/ OR ‘psychoses’/ OR ‘psychotic disorder’/ OR ‘schizophrenic disorder’/ OR ‘mental illness’/ OR ‘mental disease’/ OR ‘psychiatric’ OR ‘psychosis’/ OR ‘psychiatric disorder’/ OR ‘major psychiatric disorder’/ OR ‘major psychiatric illness’/ OR ‘brief psychotic episode’ OR ‘first episode psychosis’ OR ‘early psychosis’ OR ‘new episode psychosis’ OR ‘new psychosis’)

5. Searches 3 and 4 were then be combined (3 ‘AND’ 4)

The search strategy will be adapted for PsycINFO, EMBASE, Web of Science and Cochrane. Further search terms will be identified via descriptive terms under MeSH terms.

## Selection of studies

After conducting the initial literature search as described above in the "searches" section, two reviewers (NM and KO) will independently screen titles and abstracts, excluding studies that did not meet the eligibility criteria. Full text of potentially eligible studies will then be obtained and screened by the same reviewers. Any disagreement at this stage will be mediated through a third reviewer (GC). A PRISMA flow chart will display the articles examined at each stage, detailing the number of papers included and excluded and reasons for exclusion (online supplemental appendix 2).

## Data extraction and management

Two reviewers (NM and SA) will extract the data from included studies using a pre-piloted data collection form in order to meet MECIR standards ^36^ (online supplemental appendix 3). Any disagreement will be discussed with a third independent reviewer (ASK). The following information will be retrieved:

• General information: Study ID, reference citation, author contact details
• Methods: Aim of study, design, start and end date, duration of participation, ethical approval needed / obtained.
• Participants: Population description, setting, inclusion and exclusion criteria, method of recruitment, number of cases, number of controls, withdrawals and exclusions.
• Demographics: Age (at time sample was taken), gender, diagnostic tool, subgroups measure / reported.
• Compositional and functional outputs and information on how this data was analysed will be retrieved from all included studies. The key conclusions of the study author will be retrieved.

In case of duplicate publication of the same data, we will include the publication with most relevant data to our study question, or with the largest cohort if both are relevant. Where required information is not directly available from the studies, study authors will be contacted to request this information. Study authors will be contacted via email. In the case where no response is given a follow-up reminder email will be sent.

## Outcome data

Compositional outcomes will include alpha diversity (e.g. Shannon diversity index / Faith’s Phylogenetic Diversity), beta diversity (e.g. Bray-Curtis dissimilarity, Unifrac, Unweighted unifrac), diversity analysis (e.g. PERMANOVA), differential abundance at phylum and genus level and across taxonomic levels (e.g. ANCOM), and associations of compositional alterations with demographic and clinical characteristics of the population.

Functional outcomes will include predicted (16s) or actual (metagenomic) differential abundance of microbial genes, metabolic pathways, hormones, brain volume or function, behavioural changes, associations of functional alterations with demographic and clinical characteristics of the population.

## Risk of bias assessment

Quality assessment will be carried out using existing appraisal checklists for case-control, cohort or cross-sectional studies provided in the Newcastle-Ottawa Scale ^37^. For each study, risk of bias will be classified (high, moderate or low) by two review authors (NM and SA) independently. Data quality for each study will be recorded in a spreadsheet and a table summarising the quality of assessment/evidence. Any disagreement will be discussed with a third reviewer (ASK).

## Data synthesis and statistical analysis

Systematic Review: Studies will be grouped and analysed according to sequencing approach and key outputs, and tables will display the summary characteristics of all papers included in the systematic review. The tables will outline the following: characteristics of study populations (N, age, sex), diagnostic criteria, study design, intervention/test type, and outcomes. The outcomes refer to the difference in the diversity and composition of oral and gut microbiota between psychosis / schizophrenia groups and non-psychosis / non-schizophrenia groups.

Meta-analysis: When at least two studies reported the same outcome, the reported effect estimates will be pooled using random-effects meta-analyses. Mean difference (MD) and 95% confidence interval (CI) will be calculated, when there are standardised methods in the used scales, to assess alterations in the oral and gut microbiota in patients with psychosis compared to healthy controls. The pooled effect estimates will be displayed in a forest plot with 95% CIs. Cohen's criteria will be used to interpret effect sizes: small (0.2), medium (0.5), and large effects (0.8) ^38^. The Q and I ^2^ statistics will be used to test heterogeneity between studies ^39^. The statistical heterogeneity between studies will be assessed according to I ^2^ values; 25%, 50% and 75% will be used to indicate a low, moderate and high level of heterogeneity respectively ^38^. If high heterogeneity is observed, this will be explored using subgroup analyses. Subgroup analysis will be conducted according to study design and risk of bias assessment, and if the meta-analyses allow such an approach to be undertaken.

If the data allow for meta-analysis, a sensitivity analysis will be conducted, excluding one study at a time to assess whether specific studies have a major influence on the results. Potential publication bias will be assessed by visual inspection of funnel plots, and the asymmetry of the funnel plot will be statistically examined using Egger’s test. All analyses will be performed using Review Manager, version 5.3 (Nordic Cochrane Centre, Copenhagen, Denmark) and Stata, version 16 for Egger’s test. P<0.05 will be considered statistically significant.

## Ethics

This study is based on published data and will not include any human participants or animals; thus, ethical approval is not required. Results of this study are expected to be published in peer-reviewed journals or conference abstracts.

## Discussion

Growing evidence suggests that there are differences in the alpha and beta diversity in the microbial composition of people with psychosis and schizophrenia as compared with controls ^21, 23, 40^. A smaller number of studies explore the predicted or actual functional alterations in microbial genes or metabolic pathways associated with these differences ^41– 43^. Changes in microbiota composition in psychosis and schizophrenia have been correlated with altered brain structure and function, altered immunity, altered metabolic pathways and symptom severity. Altered metabolic pathways have, in turn, been associated with inflammatory cytokines and risk for coronary heart disease in psychosis and schizophrenia. This has been replicated in animal studies with specific behavioural patterns ^44– 48^. Changes in microbiota composition have also been identified as potential biomarkers for psychosis and schizophrenia that might aid in diagnosis. Understanding how predicted or actual functional alterations in microbial genes or metabolic pathways influence symptomatic expression and downstream clinical outcomes may contribute to the development of microbiome targeted interventions for psychosis and schizophrenia.

Our study has limitations. Given the inclusion of observational studies and the anticipated variance in study methodology and design, we anticipate large interstudy heterogeneity. Sources of heterogeneity will be further explored using subgroup analysis and meta-regression. We anticipate the number of included studies will be small. This is likely to be particularly true of those exploring the predicted alterations in functional pathways.

## Study status

Preliminary searches, piloting of the study selection process and formal screening of search results against eligibility criteria has been completed. Data extraction, risk of bias (quality) assessment and data analysis is ongoing.

## Data availability

Zenodo. Extended data for ‘Compositional and functional alterations in the oral and gut microbiota in patients with psychosis or schizophrenia: A systematic review’.

https://doi.org/10.5281/zenodo.5501952 ^49^

This project contains the following underlying data:

• Appendix 1: Completed PRISMA-P checklist
• Appendix 2: PRISMA flow diagram for studies of oral and gut microbiome in psychosis or schizophrenia
• Appendix 3: Data collection form

Data are available under the terms of the Creative Commons Zero "No rights reserved" data waiver (CC0 1.0 Public domain dedication).

## Contributors

KO is the guarantor of this systematic review, initiated this research and designed the systematic review protocol. NM, AK and GC contributed to the design and revision of the systematic review protocol. NM, KO and GC completed the pilot literature search and the formal selection of studies. NM, SA and AK will conduct the data extraction, evaluation of risk of bias and quantitative synthesis. NM and KO will draft the manuscript. All the authors will be involved in result interpretation. All the authors contributed to the review and revision of the manuscript and approved the publication.
