# Supplementary material for: Compositional and functional alterations in the oral and gut microbiota in patients with psychosis or schizophrenia: A systematic review
Source: HRB Open Res. 2021 Sep 29;4:108. [Version 1] doi: 10.12688/hrbopenres.13416.1 (PMC8634050; doi:10.12688/hrbopenres.13416.1)
Supplement: Peer review history

# Peer review: Reviewer response for version 1

**DOI:** 10.21956/hrbopenres.14618.r30846

**Published:** 2021-11-30

**Authors:** Melanie Focking

**Affiliations:** 1 Department of Psychiatry, Royal College of Surgeons in Ireland, Dublin, Ireland

The microbiome is an interesting and rapidly growing area of research and its importance for human health is becoming more and more obvious. How the gut microbiome affects the mammalian brain, especially in mental disorders is an area of great interest currently. Different teams using different approaches, both in men and mice have been published in this field. The aim of the current project is to streamline this information, by undertaking a systematic review and meta-analysis of relevant data to further the understanding how changes in microbial genes or metabolic pathways influence symptomatic expression and clinical outcomes. It is hoped that the information gained will aid in development of microbiome targeted interventions for psychosis.

 The manuscript study protocol submitted gives a great background to the field and describes the need for a systematic review and meta-analysis. The authors are very clear about their search strategy and have already proven that the keywords used are giving good results. In addition, they have already used the PRISMA template (the golden standard for systematic review) for their search and have screened the articles obtained and funnelled down the list to 21 papers/studies to be included. The hypothesis of their work is well described and the authors seem very realistic about the outcome. To me, this looks very well thought through and I have no doubt that despite the small number of studies included, some good results can be expected that will further the field.

 While I am very enthusiastic about the study and the information that will be gained, the authors do propose to include findings from animal studies. Not much information is provided in the manuscript so far but it would be helpful to the reader if the authors state how they are planning to integrate this information and if they anticipate this could cause some problems.

 Overall, a very well described manuscript and a study to watch out for.

Is the study design appropriate for the research question?

Yes

Is the rationale for, and objectives of, the study clearly described?

Yes

Are sufficient details of the methods provided to allow replication by others?

Yes

Are the datasets clearly presented in a useable and accessible format?

Yes

Reviewer Expertise:

Psychotic disorders, clinical high risk of psychosis, first episode psychosis, proteomics, biochemistry

I confirm that I have read this submission and believe that I have an appropriate level of expertise to confirm that it is of an acceptable scientific standard.

# Peer review: Reviewer response for version 1

**DOI:** 10.21956/hrbopenres.14618.r30530

**Published:** 2021-10-14

**Authors:** Belinda R. Lennox, Monty Lyman

**Affiliations:** 1 University of Oxford, Oxford, UK; 2 Department of Psychiatry, University of Oxford, Oxford, UK

This protocol for a systematic review is detailed, thorough and well placed to answer a clinically relevant and interesting research question. The methods – from assessing risk of bias to statistical analysis – fulfil PRISMA criteria and are clearly described, enabling replication. The search terms strike a good balance of being both broad enough to identify many papers but also targeted enough to be relevant to answering the research question.

 We have a few minor suggestions for the authors:

 1) The introduction should include clear definitions of both compositional and functional alterations. While these are indirectly described throughout the paper - and fleshed out in the 'Outcome data' section - these should be clearly defined at the start of the paper.

 2) In the introduction, it states that 'psychosis is associated with an array of mental illnesses including schizophrenia, schizoaffective disorder, delusional disorder, bipolar disorder and depression', however only schizophrenia features in the search strategy. If other disorders such as bipolar disorder are not included, it would be helpful if the rationale for this is explained in the paper.

 3) The sentence ‘In fact, gastrointestinal diseases are the third leading cause of natural deaths in schizophrenia’ in the introduction is arguably extraneous to the argument and direction of the paper. The authors of the reference supporting this sentence admit that their paper does not take into account the influence of antipsychotics on gastrointestinal diseases, including GI dysmotility and clozapine-induced bowel obstruction. Perhaps this statement could be omitted?

 Aside these minor suggestions to the introduction, we believe that this is an excellent study protocol and we look forward to reading the results on completion of the study.

Is the study design appropriate for the research question?

Yes

Is the rationale for, and objectives of, the study clearly described?

Yes

Are sufficient details of the methods provided to allow replication by others?

Yes

Are the datasets clearly presented in a useable and accessible format?

Not applicable

Reviewer Expertise:

Academic Clinical Fellow in Psychiatry, University of Oxford. Studying the relationship between the gut microbiome and mental health.

We confirm that we have read this submission and believe that we have an appropriate level of expertise to confirm that it is of an acceptable scientific standard.
